# Supplementary material for: Are carbon emissions trading and green financial instruments synergistic? -Comprehensive quantitative research based on content analysis
Source: PLoS One. 2024 Mar 7;19(3):e0298601. doi: 10.1371/journal.pone.0298601 (PMC10919601; doi:10.1371/journal.pone.0298601)
Supplement: S1 Data — (ZIP) [file pone.0298601.s001.zip › Data/地级市Z_F工作报告.zip!/░▓╗╒/╨√│╟╩╨/└┤╫╘╣1⁄2╓┌║┼í╛┬φ┐╦╩2╛▌═°í┐_2016.docx]

政府工作报告——2016年1月13日在宣城市第三届人民代表大会第七次会议上 市长 韩 军

各位代表：

现在，我代表市人民政府，向大会报告政府工作，请予审议，并请市政协委员和列席会议的同志提出意见。

一、2015年及“十二五”经济社会发展跨上新台阶

刚刚过去的一年，全市上下在市委的坚强领导下，认真贯彻中央和省决策部署，坚持“11331”发展思路和“好为标准、快字当先”工作导向，全力推进“六年并进、六城同创”，主动作为，攻坚克难，较好地完成了市三届人大六次会议确定的目标任务，实现了新常态下的新发展。

初步测算，完成地区生产总值971.6亿元，同比增长8.2%。财政收入188.9亿元，增长8%。固定资产投资1283.4亿元，增长12.6%。社会消费品零售总额422.5亿元，增长12.6%。进出口总额18.5亿美元，增长9.6%。城镇居民人均可支配收入28700元，增长9%左右；农村居民人均可支配收入12400元，增长10%左右。

（一）全力促进实体经济发展，夯实稳定增长基础。着力创新服务方法。全面落实稳增长政策措施，强化经济工作督导和运行调度，开展“助企解困”行动和“精准帮扶”活动。设立市中小企业应急转贷周转资金，开展应急转贷服务144次，解决企业调头资金9.1亿元。坚持和完善市投融资管理委员会工作制度。设立首期5亿元的产业投资母基金，同步设立三支子基金。建立PPP滚动项目库，首批2个PPP项目签约。旌德成功实施全国首个“担保+PPP”模式项目。着力畅通金融血脉。大力推进“4321”风险分担机制，开展银政担创新合作业务426笔、18.4亿元；“税融通”贷款发放18笔、0.8亿元。实施企业上市“翱翔计划”，3家企业A股首发上市，7家企业“新三板”挂牌，24家企业在地方性场外市场挂牌。市国投公司新增融资到位资金70.7亿元。全社会融资总量130多亿元，其中直接融资30.7亿元。着力扩大有效投入。398个列入省亿元以上重点项目完成投资466亿元。争取中央预算内投资和省补助资金13.1亿元、专项建设基金13.3亿元。谋划储备“十三五”亿元以上项目2268个，计划投资8831亿元。强化项目建设保障。清理“三类”土地1.32万亩，清理处置率达90%，居全省前列。开展市区集中征迁拆违行动，征地2452亩，征迁950户、19万平方米，拆除违法建设3.3万平方米。

（二）加快结构调整步伐，推动经济转型升级。在全省率先出台调结构转方式促升级行动方案，276个项目列入省“调转促”重点项目库。工业经济实现新突破。汽车和装备制造、食品医药、新材料三大产业产值占比达47%，提高5.2个百分点。有色金属压延、钢材和水泥三大传统行业占比降至10.9%，下降1.7个百分点。高新技术产业增加值增长16%。战略性新兴产业产值增长22%以上，居全省第6位。宁国经开区核心基础零部件产业集聚发展基地入选全省首批战略性新兴产业基地。实施“双百”工业重点项目，项目新开工率、投产率达100%。司尔特年产90万吨新型复合肥和25万吨硫铁矿制酸项目、海螺年产160万吨水泥粉磨系统项目开工建设。新增规模工业企业206户，居全省第4位。规模工业企业利润居全省第5位。获得全省首个创新驱动助力工程示范市称号。新增省级“专精特新”企业10户，新认定省级工业设计中心3家、企业技术中心6家、工程技术研究中心9家。安徽宏宇竹业科技股份有限公司荣获国家科学技术进步二等奖，我市科技成果30年后再次跻身国家奖行列。宁国成为全省唯一跻身国家知识产权示范市的县级市。新增中国驰名商标3件、省著名商标59件、安徽名牌产品13个。文化旅游产业加快提升。全年文化产业增加值增长12%；游客接待量突破2000万人次、旅游总收入增长18%。中国宣纸博物馆建成并对外开放。胡开文墨业公司入选第六批国家文化产业示范基地，8家企业入选安徽民营文化企业100强。良才墨业“御制铭园图”套墨获巴黎首届国际艺术博览会金奖。宁国获评美丽中国十大最美城镇。泾县成为国家级休闲农业与乡村旅游示范县。4家景区通过4A级景区景观质量评估。完成国家级全域旅游示范区、省级旅游度假区创建申报。在全省率先启动研学旅行基地建设，云岭新四军军部旧址纪念馆入选全省首批十大研学旅行基地。成功举办第七届文房四宝文化旅游节、三月三敬亭山民俗风情月、第四届宁国山核桃文化节等节庆活动，开展“《百家讲坛》名家评宣城”系列活动。敬亭山旅游度假区建设加快，韩愈文化园、敬亭诗径、太白独坐楼建成开放。谢朓楼修复完成，广教寺双塔塔院山门复建工程完工。敬亭湖宾馆建成开业。现代服务业发展加快。出台促进健康产业发展实施方案，编制健康养老产业示范基地建设方案。爱晚工程·国家养老示范基地等项目加快推进。大力发展商贸流通产业，实施7个省级市场体系建设项目，稳步推进宁国、广德农产品流通试点县建设。加快电子商务发展，5个县电商发展指数跻身全省县域十强。绩溪成功搭建“上街去”区域性第三方电商平台；宁国成立全省首个农村淘宝项目服务中心，淘宝特色中国·绩溪馆和1号店特产中国·广德馆相继成功上线。现代农业稳步推进。粮食总产128.7万吨，增长5.2%。新增耕地流转面积11万亩，流转率达42%。郎溪获批国家级现代农业示范区和全国首批家庭农场监测县。宣州洪林农业科技园区成为国家级农业科技园区。新增农民专业合作社269个、家庭农场979个。农产品加工产值650亿元，增长12%。在全省率先建成市县生猪定点屠宰在线监控平台，市农产品质量安全检测中心建成运行。宣州成为国家农产品质量安全创建试点县。实施特色林业产业三年发展规划，新增国家级林业重点龙头企业2家，林业总产值420亿元左右。推进“放心粮油”和“主食厨房”工程建设，认定示范店、经销点和示范企业48家。完成危仓老库维修改造任务。

（三）全面深化改革开放，激发发展活力动力。重点改革有力推进。年度74项改革重点任务全面完成。政府权责清单和涉企收费清单制度全面实施，市级政府权力清单运行平台试运行；行政审批项目减少到98项，审批环节进一步压缩，政务服务提速提效明显。“三证合一、一照一码”改革实现县市区全覆盖，新增民营企业4929户、个体工商户1.95万户。

完成不动产统一登记职能整合和机构设置。农村土地承包经营权确权登记颁证工作进展顺利，8个试点村启动农村集体产权制度改革。宣州区被确定为全国农民住房财产权抵押贷款试点县。深化户籍制度改革，全面放开落户政策，发放居住证1.1万张。启动宣州区社区综合体制改革试点。完成机关事业单位工资改革，实施市县机关公务用车制度改革。招商合作全面深化。坚持领导带头，量化领导干部外出招商、推进项目落地的时间和任务。围绕重点区域、重点目标，广泛开展投资合作推介活动；突出产业招商，探索市场化招商，加强与央企、知名民企对接，新引进亿元以上项目194个，中兴新材料、北斗应用、晶科光伏、中粮肉食产业链基地等一批项目签约。亿元以上项目实际到位省外资金730亿元，增长21.8%，总量居全省第3位。外向型经济平稳发展。宣城海关、国检监管查验场所投入运营，宣城“无水港”项目获省政府批准，广德开通全省首个县域报关点。新增进出口企业100家、进出口额1000万美元以上企业28家。实际利用外资7.96亿美元，增长15.4%，增幅居全省第1位。实际对外投资首次突破1亿美元，居全省第3位。

（四）加强中心城市建设，提升城市综合承载力。出台进一步加快皖苏浙交汇区域中心城市建设的意见。夯实中心城区产业基础。强化市区一体发展，集聚发展合力。宣城经济技术开发区狠抓工业和项目两个重点，新签项目81个，竣工投产工业项目20个，亿元以上项目实际到位省外资金43.8亿元，实际利用外资8195万美元。完成固定资产投资43.2亿元，世行项目“八路一桥一厂”工程加快推进。宣城现代服务业产业园区建设加快，新塘羽绒产业园新落户企业25户。旅游商品博览城一期、联邦物流大市场一期建成运营。宣州区坚持强工富区，重点推进“两区四园”建设，新签项目190个，新建、续建重点项目211个。持续加大基础设施建设。新一轮城市总体规划报省待批，开展了环南漪湖地区概念性总体规划、城市控制性详细规划通则等编制工作，编制完成城市水系等7项专项规划。续建和新建中心城区城建项目167个，完成投资121.1亿元，其中城市基础设施投资58.6亿元。体育馆投入使用，图书馆、青少年综合实践基地、国防动员训练基地建成交付，规划馆、博物馆正在布展。向阳大道一、二标段和陵阳路一期等建成通车，宣水路、有容路、花园路3条“断头路”相继打通。建成明镜湖公园、泥河景观带、清溪河景观带等7个公园和12处街头游园，九曲公园基本建成，植物园建设加快推进。实施陵阳路、宝城路等11条道路绿化提升。巩固老旧小区综合整治成果，实施消防安全专项整治，新建城市消防站4座。完成市区4条主要道路和2个出入口沿街建筑立面整治。关庙水库主体工程、敬亭圩除险加固等工程完成。宣城历史上单项投资规模最大的准东—华东（皖南）±1100千伏特高压直流输电工程古泉换流站项目正式落地，500千伏广德智能变电站等11项重大电网工程顺利投运。率先建成“全光网市”，城镇实现光网全覆盖，农村覆盖率超过90%。加快美好乡村建设。扎实推进112个省级中心村建设，继续开展“三线四边”环境治理，农村垃圾处理常态化水平进一步提升，在全省率先通过农村生活垃圾治理省级验收。旌德成为全省首批美好乡村建设整县推进试点县。强力推进交通建设会战。完成交通建设投资81亿元。宣南铜、宁千高速公路和宣泾路、G205泾南段等一级公路建成通车，宣狸、溧广高速公路和S214郎溪段等项目加快推进，宁绩高速鸿门互通、水阳江航道综合整治和宣州综合码头等项目开工建设，完成公路路面改善260公里，建成村村通延伸工程270公里。有效推进“六城同创”。全国文明城市创建“基础年”各项任务圆满完成。创成国家森林城市、全省首个中华诗词之市和省级卫生城市。入选全国第二批生态文明先行示范区。全国双拥模范城创建通过验收，国家园林城市即将授牌。泾县顺利通过国家生态县考核验收，广德、旌德通过技术评估。国家生态市创建全部达标，环保模范城市创建进入迎检。生态环境持续优化，大气污染防治目标完成进度居全省第2位。城市知名度、美誉度空前提升。圆满承办我市有史以来规格最高、规模最大的全国2015森林城市建设座谈会。第七届文房四宝文化旅游节综合成效创历届之最，皖南示范区7市首次联手参加。成功举办首届综合性竞技体育运动会，承办全国山地自行车冠军赛等重大赛事。城市形象片《美在宣城》喜获2015美国亚特兰大短片节“聚光灯短片大奖”。电视剧《文房四宝》在安徽卫视播出。在美国米尔肯研究所发布的中国最佳表现城市榜单、上海三家社会机构评选的中国城市竞争力排行榜和人民日报社开展的地市治理能力测评排名中，我市均居安徽第2位。

（五）切实保障改善民生，维护社会和谐稳定。民生保障稳步提升。投入资金36.6亿元，完成33项民生工程和市政府10件民生实事。新增城镇就业4.6万人，登记失业率3.23%。城镇职工基本医疗保险政策范围内住院费用报销比例达84%，为全省最高。强化社会救助，城乡特困人员实现应保尽保，启动市救助站和市精神病人福利机构建设。市残疾人康复中心建设进展顺利。新开工各类保障性住房和棚户区改造安置住房13970套，基本建成15178套，完成农村危房改造14600户。全面推进精准扶贫，预计减少贫困人口2.6万人。社会事业协调发展。出台柔性引进市外人才办法，共引进紧缺高层次人才59人、人才团队7个，入选省“115”产业创新团队2个。新改扩建公办幼儿园12所。261所义务教育学校标准化建设任务全部完成。实施基础教育信息化三年建设计划，中小学宽带接入率、校园网建成率均达100%。绩溪、旌德通过义务教育发展基本均衡县省级评估。普通高考本科总达线率、一本达线率分别提高到62.5%和24.8%，进入全省领先行列。职业教育办学质量不断提升，职业技能大赛总成绩居全省第4位，对口升学考试录取人数和录取率位居全省第一。巩固完善基层医改和县级公立医院综合改革，全面实施城市公立医院改革。计划生育工作继续保持全省领先，“单独两孩”政策平稳实施。106个公共文化场馆免费开放，完成254个20户以下已通电自然村广播电视“村村通”建设任务，农村公益电影放映8000多场、送戏进村704场，更新农家书屋图书8万余册。中国书法城、中国摄影之乡申创工作有序推进。宁国创成国家级摄影创作基地。宣州、泾县、绩溪、旌德获批皖南(县域)国家体育产业基地。深入开展第二次全国地名普查。工商联、外事侨务、对台、民族宗教、档案、地方志、防震减灾、机关事务等工作得到加强，全面完成妇女儿童“两个规划”中期重点目标任务，工会、共青团、老龄、残疾人、慈善福利和红十字事业进一步发展。社会治理得到加强。建成市政务云计算中心，市社会服务管理信息化平台试运行。完成社区换届工作。开展机关集中学法月活动，加强公共法律服务体系建设，扩大法律援助覆盖面，全面完成“六五”普法目标任务。推进信访制度改革，实行信访责任制和责任追究制，深化领导干部开门接访制度，全力化解信访积案，信访形势持续向好。严格落实安全生产责任制，安全事故起数、死亡人数分别下降13.9%和4.6%，未发生较大及以上事故。全力争创食品药品安全城市，泾县、广德成功创建省级食品安全示范县。加强反恐应急处突能力建设，完善社会治安防控体系，依法打击各类违法犯罪活动，群众安全感满意度、政法机关和政法队伍执法工作满意度均居全省第一。圆满完成征兵任务和退役士兵安置工作，国防动员、国防教育、人民防空工作进一步加强。

（六）加强政府自身建设，提升履职尽责能力。扎实开展“三严三实”专题教育。认真贯彻落实中央八项规定和省、市委“三十条”规定，扎实开展查处群众身边“四风”和腐败问题专项工作。严格落实重大行政决策程序规定，完善政府法律顾问制度。启动地方政府规章立项工作。自觉接受人大法律监督、工作监督和政协民主监督，落实市政府负责同志领衔办理制度，办理人代会议案2件、人大代表建议80件、政协提案（建议）205件。完善百姓热线、百姓问政等工作机制，督促解决群众反映的热点问题。强化行政监察和审计监督，深化预算执行审计和领导干部经济责任审计，加强政府投资建设项目审计，审计节约资金5.4亿元。对市直91个单位“三公”经费、津补贴发放等开展专项审计核查。规范和加强政府信息公开。强化公共资金管理，实施市直单位实有资金账户财政统管。深化市区公共资源交易管理体制改革。实行“一单清”和工作落实登记积分制度，出台对庸政懒政怠政行为问责暂行办法，强化对决策部署落实的督查问责，促进政令畅通。

各位代表！2015年目标任务的顺利完成，标志着“十二五”发展的圆满收官。回望过去的五年，宏观经济形势复杂多变，改革发展稳定任务艰巨繁重，全市上下咬定目标、沉着应对，抢抓机遇、开拓奋进，经济社会发展取得了令人瞩目的成就。

过去五年，是经济持续较快发展、综合实力大幅提升的五年。主要指标基本实现“五个翻番、一个高于、一个同步、一个确保”的规划目标。地区生产总值接近1000亿元，是2010年的1.85倍，在全省前进两个位次；服务业增加值是2010年的1.88倍；财政收入是2010年的2.2倍；规模工业增加值是2010年的1.56倍；五年累计完成固定资产投资4848亿元，年均增长21%。城镇和农村居民人均可支配收入均居全省第5位，年均增长12.5%和13.5%，分别高于生产总值增幅1.5个和2.5个百分点，城镇居民人均可支配收入增幅连续5年保持全省前3位。“十二五”节能减排任务全面完成。

过去五年，是三次产业协调发展、经济结构不断优化的五年。三次产业结构由16.8∶47.2∶36调整为13∶50.5∶36.5。新型工业化进程加快。工业化率由2010年38.4%提高到44.5%。新增规模工业企业788户，总数达1389户，居全省第5位。实现规模工业企业利润和民营企业税收“两个百亿”突破。中鼎集团成为全市首个营业收入超百亿企业，并入围全国民营企业500强。战略性新兴产业产值、高新技术产业增加值占比分别比2010年提高15和14个百分点。培育发展高新技术企业166家，建立省级以上创新平台95个。单位工业增加值能耗累计下降46%，以年均增长1.46%的能耗支撑了规模工业年均15%的发展速度。文化旅游产业加速发展。启动实施文化旅游产业“八大工程”三年行动计划，新增4A级以上景区8家，龙川景区创成5A景区，创建国家级休闲农业与乡村旅游示范县3个、示范点4个、精品线路1条。游客接待量、旅游总收入年均增长16%和18%，文化产业发展指标连续两年位列全省第3名，文化产业增加值占比达4.2%。城市商业品质提升，国购广场、星隆国际广场等一批大型城市综合体和商业项目建成开业。县域电商发展居全省前列，绩溪、广德被商务部列为电子商务进农村综合示范县。现代农业稳步发展。创成国家级农业产业化示范区2个、省级3个，国家级现代农业示范区1个、省级5个，农产品加工产值是2010年的2.5倍。

过去五年，是项目建设提质提效、基础设施明显改善的五年。列入省亿元以上重点项目开工率、竣工率和投资完成率连续五年位居全省前列。城镇化率达51.1%，较“十一五”末提高7.8个百分点。中心城区累计完成城建投资665.9亿元，其中城市基础设施及公共建设投资308.5亿元。建成区面积扩展到55平方公里。交通建设会战取得突破性进展，完成投资310亿元，是“十一五”时期的5倍。新增高速公路220公里，是“十一五”末的2倍多，进入全省前四位；建成一级公路275公里，改善路面1100公里以上，建成村村通延伸工程1200公里以上，公路路况水平由全省落后跃居第2位。完成芜申运河宣城段整治和定埠港区工程，新增三级航道10公里。完成铁路建设投资93.2亿元，合福高铁开通运营，皖赣铁路芜宣段扩能改造、杭黄铁路、商合杭铁路开工建设。完成水利建设投资66.2亿元，水阳江开卡工程顺利完工，农村水利基础设施水平和城市防洪排涝能力显著增强。完成电网基建投资37.7亿元，投产110千伏以上线路、变电容量较“十一五”时期增长2倍多。

过去五年，是重点改革不断深化、对外开放深入推进的五年。扎实推进政府职能转变、简政放权、放管结合、优化服务。开展省直管县体制改革和经济发达镇改革试点。有序推进“营改增”试点，兑现补贴6.8亿元。民营经济迅速发展，新增民营企业1.75万户、个体工商户7.6万户，民营经济占生产总值比重由2010年65%升至71%，居全省第1位。宣城经济技术开发区、宁国经济技术开发区成功升格为国家级开发区。宣城海关、出入境检验检疫局开关开检。成功加入南京都市圈。对外贸易额比“十一五”时期增长3.3倍。招商合作成效显著，累计引进亿元以上项目1380个，实际到位省外资金2820亿元，位居全省前列。实际利用外资28亿美元，是“十一五”时期的4倍。

过去五年，是城市创建喜讯不断、品牌影响力逐步彰显的五年。成功创成省级文明城市，并获得全国文明城市创建提名。在全省率先启动创建国家生态市，创成全省首个省级生态市。环境保护工作连续四年保持全省先进行列。顺利通过“中国文房四宝之乡”复评并更名为“中国文房四宝之城”，文房四宝文化旅游节被评为“美丽中国·最具品牌价值文化艺术节”。实施森林增长工程，新造林75万亩，新建绿色长廊442.7公里，森林覆盖率达58.03%，比“十一五”末提高近3个百分点。全力推进中心城区“一山一湖三河十园”和街头游园建设，新增绿化面积778.6公顷。城市绿地率达36.4%，人均公园绿地面积13.5平方米。城市精细化、数字化、科学化管理进一步加强，城市文明程度进一步提高，山青水秀、山水相依的城市风貌进一步彰显，市容市貌和人居环境显著改善。社会各界对宣城的认同感、赞誉度有新提升，宣城的影响力逐步扩大。

过去五年，是社会事业全面发展、群众获得感显著增强的五年。财政累计投入民生工程资金141.7亿元。五年城镇新增就业25万人。城乡居民社会保险连年超额完成省定任务，城乡低保标准、农村五保标准比2010年分别提高69.4%、121.8%和110%。新增社会办养老福利机构15所，新改扩建农村敬老院93所、公办社会（儿童）福利院7所。开工各类保障性住房7.75万套。改造农村危房6.7万户，解决农村96.2万人饮水不安全问题。新建公办幼儿园97所，新增学位15520个。持续推进中心城区学校建设，新改扩建中小学校17所，新增学位16450个，大班额现象得到缓解。合工大宣城校区开学并实现满建制办学。医药卫生体制综合改革不断深化，建立覆盖城乡的基本医疗服务网络。市县乡村四级公共文化服务体系基本建成。27个村成为省级农民文化乐园试点村。参加省十三运会成绩上升四个位次，取得历史性突破。公共文化场馆等级率和人均体育场地面积均居全省第3位。法治宣城、平安宣城建设不断加强，市政府连续四年被省政府评为安全生产目标管理先进单位，群众安全感满意度、政法机关和政法队伍执法工作满意度连续五年位居全省前列。

各位代表！五年的砥砺奋进，五年的辉煌成就，奠定了我市全面建成小康社会的坚实基础，开启了宣城加速崛起的崭新局面。这是省委、省政府和市委坚强领导的结果，是市人大、市政协监督支持的结果，是全市人民团结奋斗的结果。在此，我谨代表市人民政府，向全体人大代表、政协委员，向全市广大工人、农民、知识分子、干部和离退休老同志，向驻宣解放军指战员、武警官兵和政法干警，向各民主党派、工商联、各人民团体和社会各界人士，致以崇高的敬意！向关心支持宣城建设的中央、省驻宣单位，向在我市创业奋战的投资者、建设者，表示衷心的感谢！

在充分肯定成绩的同时，我们也清醒认识到，前进道路上还存在不少困难和问题。一是中心城市规模不大、实力不强、质量不高的状况没有得到根本改观，竞争发展压力大，区域中心城市建设任重道远。二是经济结构不优，产业层次不高，高新技术产业、战略性新兴产业基础薄弱；企业创新能力不强，大多缺乏核心技术和有效应对市场风险能力。三是重大项目、创新性项目、竞争力强的项目少，承接产业转移项目多为异地扩能项目，支撑加快发展的动力不足。四是财政收支压力加大，潜在金融风险隐患增多；民生工程和社会事业供求不均衡，一些新的因素影响社会和谐稳定。同时，少数部门服务不到位、监管不到位，个别干部负责担当、主动作为意识不强，“四风”问题仍不同程度存在。对此，我们将持续高度重视，认真研究，加力加快解决。

二、奋力谱写“十三五”宣城发展新篇章

“十三五”期间，是宣城提前1—2年全面建成小康社会的决战期、调转促的关键期、皖苏浙交汇区域中心城市的形成期、扶贫任务的攻坚期和文明创建的提升期。我们将主动适应和引领经济发展新常态，坚持发展第一要务和五大发展理念，以加快调结构转方式促升级为主旋律，深入实施生态立市、工业强市、文化名市“三大战略”，努力实现经济规模和质量、现代服务业、中心城区发展、重要基础设施、体制机制创新“五个新突破”，提前全面建成小康社会，皖苏浙交汇区域中心城市建设取得阶段性成果。

根据市委的建议,市政府编制了“十三五”规划《纲要》（草案），提请本次大会审议。综合考虑，今后五年全市经济社会发展的主要奋斗目标是：

——经济保持中高速增长。到2020年，地区生产总值、财政收入分别实现1500亿元、260亿元，冲刺1600亿元、280亿元。

——发展方式转变取得实质性进展。创新能力明显提高，研发经费支出占地区生产总值比重提高到2.5%，科技进步对产业增长的贡献率提高到65%。三次产业结构进一步优化。

——重点改革取得决定性成果。使市场在资源配置中起决定性作用和更好发挥政府作用。在政府服务、资源要素市场化配置、生态文明、农村综合配套等重点领域和关键环节的体制机制改革取得突破。

——生态环境质量进一步改善。绿色发展理念贯穿经济社会发展全过程，生产空间、生活空间、生态空间布局科学，生态文明发展模式基本形成，建设全国生态文明先行示范区。

——人民生活质量和水平持续提高。城乡居民收入增幅高于地区生产总值增幅。城乡收入差距进一步缩小。基本公共服务水平进一步提高，社会保障体系趋于完善。提前实现整体脱贫目标。

——社会文明程度大幅提升。社会主义核心价值观深入人心，公民文明素质和社会文明程度明显提高。覆盖城乡、标准化均等化的现代公共文化服务体系基本建立，依法治市全面推进，法治宣城建设取得明显成效。

实现上述目标任务，我们将聚力打造“四个特色之城”：

（一）聚力打造现代产业之城。以加快“调转促”为总抓手，构建以战略性新兴产业为先导、先进制造业为主体、现代服务业为支撑、现代农业为基础的富有活力的产业之城。加快建设适应市场需求的新兴制造业基地。以战略性新兴产业集聚发展基地建设为突破口，建成10个产值超百亿元基地。实施“千企升级计划”，加快传统产业改造提升，力争技改投入达到2000亿元。到2020年，汽车和装备制造业成为首个超千亿产业。加快建设国家级全域旅游示范区。实施全域旅游发展战略，推进皖南国际文化旅游示范区建设，加快实施文化旅游产业“八大工程”，提升“山水诗乡、多彩宣城”城市品牌。到2020年，新增5A级景区1—2家、4A级10家，创建全国休闲农业与乡村旅游示范县2个、省级旅游度假区5个，实现游客接待量4500万人次、旅游总收入350亿元，旅游经济总量跻身全省前六位。加快建设现代服务业集聚发展区。适应消费升级趋势，大力发展电子商务、互联网金融、信息服务、现代物流、健康养老养生等产业。到2020年，现代服务业增加值占服务业比重超过60%，培育15个省级现代服务业集聚区，申创国家级现代服务业集聚区、电子商务示范基地。加快建设现代生态农业示范市。以发展现代生态农业为重点，加快转变农业发展方式,走产出高效、产品安全、资源节约、环境友好的现代农业发展之路。到2020年，创建省级现代生态农业示范县7个、示范乡镇30个、示范主体100个，现代生态农业发展指标位居全省前列；农产品加工产值达1200亿元。

（二）聚力打造综合枢纽之城。充分发挥区位优势，扩大开放合作，加速人流、物流、资金流、信息流向宣城聚集。建设区域综合交通枢纽。完成交通建设投资300亿元，实现县县通高速，加快区域联网联通，构建高速畅通网，继续加快一级公路等国省干道建设，实现“市县一小时、县乡半小时”的通达目标。加力推进铁路建设，形成以2条普速铁路为基础、5条快速铁路为骨架、宣城为枢纽节点、县县通高铁的区域铁路格局。加速形成“干支成网、水陆联运、转换便捷、畅通高效”的综合交通运输体系。提升中心城市承载能力。加快中心城区建设，强化城市规划、建设和管理，完善城市功能，优化城区业态，提高城市品位，城市建设投资600亿元，力争750亿元。探索开展行政区划调整，支持广德撤县设市。加快农业转移人口市民化进程，推动人口集聚，到2020年，全市常住人口城镇化率达到58%，户籍人口城镇化率达到46%，中心城市首位度进一步提升。构建开放合作新格局。主动参与“一带一路”、长江经济带建设，深化与上海、南京、杭州等都市圈合作。建设对外开放新平台，加快宣城、宁国两个国家级开发区发展步伐，大力推进宣城现代服务业产业园区和宣州、广德开发区转型发展，建设宣城“无水港”，申请设立内陆口岸，积极推进申报安徽皖东南保税物流中心和市区公用型保税仓库，培育优势产品出口集聚区。积极发挥海关、出入境检验检疫局作用，推进通关一体化和贸易便利化建设。

（三）聚力打造山水生态之城。创成国家环保模范城市，在全省率先建成国家生态文明建设示范区。不断提升国家园林城市、森林城市建设水平，形成河流水系、自然山体、生态绿地和城市空间有机连接的绿色生态安全格局。全面实施城市水生态建设三大工程，实现“引得来、蓄得住、水质好”的目标。强化水、大气、土壤污染防治，确保消除劣V类水体，城镇集中式饮用水源水质稳定达标，到2020年，重点水体水质优良率提高到85%以上；到2017年，可吸入颗粒物浓度比2013年下降23%以上。持续推进美丽乡村建设，加快农村基础设施建设，探索中心村建设管护机制，实现中心村建设和农村环境整治全覆盖，着力打造“美丽中国”建设先行区。

（四）聚力打造平安幸福之城。坚持就业优先，以创业带动就业，新增城镇就业15万人，城镇调查失业率控制在5%以内。健全社会保障体系，实施全民参保计划，基本实现法定人员全覆盖。坚决打赢脱贫攻坚战，确保2016年建档立卡贫困人口全部脱贫，2017年贫困村全部出列。加快发展普惠性学前教育，全面改善义务教育薄弱学校基本办学条件，推动义务教育均衡发展。全面普及高中阶段教育。提高教育教学质量。规划建设职教园区，发展提升职业教育。推进学习型城市建设。以培育和践行社会主义核心价值观为根本，以提升城市文明程度为目标，创成全国文明城市。完善公共文化服务体系，开展群众喜闻乐见的文体活动。深化医药卫生体制综合改革，加快建立全覆盖、保基本、多层次、可持续的基本医疗卫生制度，促进基本公共卫生服务均等化，形成高质量的城市居民“15分钟医疗卫生服务圈”和“15分钟健身圈”。深入推进法治宣城、平安宣城建设，全面建成覆盖城乡居民的公共法律服务体系。创新社会治理方式，提升社会治理水平，确保社会和谐有序、充满活力。

三、努力开创2016年各项工作新局面

2016年是全面建成小康社会决胜阶段的开局之年，也是推进结构性改革和实现脱贫的攻坚之年。做好今年工作，以优异成绩为本届政府划上圆满句号，具有十分重要的意义。

今年政府工作的总体要求是：全面贯彻党的十八大和十八届三中、四中、五中全会及中央、省经济工作会议精神，认真贯彻“四个全面”战略布局和五大发展任务要求，牢固树立和贯彻落实创新、协调、绿色、开放、共享五大发展理念，主动认识、适应、引领经济发展新常态，坚持稳中求进工作总基调，坚持“好为标准、快字当先”工作导向，认真落实全市经济工作会议提出的“2365”工作总体安排，即坚持“11331”发展思路和“六年并进、六城同创”活动“两个不变”，紧盯提前全面建成小康社会、提前完成精准脱贫任务、皖苏浙交汇区域中心城市建设取得阶段性成果“三大目标”，聚焦稳增长、调转促、结构性改革、城市建设、农业农村、民生稳定“六个重点”，提升把握大势、推进落实、依法行政、党建保障、廉洁自律“五个能力”，确保“十三五”发展良好开局。

建议全市经济社会发展的主要预期目标是：地区生产总值增长8%左右，财政收入保持基本同步，规模工业增加值增长7.5%左右，服务业增加值增长9%左右，固定资产投资增长10%左右，社会消费品零售总额增长11%左右，进出口总额保持正增长，城镇居民人均可支配收入与经济增长同步，农村居民人均可支配收入增幅高于城镇居民收入增长水平，新增城镇就业3.3万人，完成省定节能减排任务。以上为确保性指标，在执行中我们力争完成得更好。

全年工作重点做好以下八个方面：

（一）千方百计扩大有效投入。强化项目谋划争取。围绕调转促“485”行动方案，加大战略性新兴产业、城镇基础设施、棚户区改造等项目谋划力度，建立主导产业滚动推进项目库，力争更多项目纳入国家和省规划，争取纳入省重点项目库项目450个以上。加快重点项目建设。继续开展“项目建设提质提效年”活动，实施重点项目440个以上，完成投资310亿元以上。列入省重点项目投资完成率、开工率、竣工率继续保持全省前列。推广运用PPP等模式，引导和鼓励社会资本参与公共服务供给，确保阳德路及周边地块改造、涉铁市政等项目开工建设。强化项目建设保障。坚持完善领导联系重点项目制度，严格落实“四督四保”制度，加强分层分级分类调度，确保项目推进有力有效。加力争取上级各类专项资金，社会融资总量增幅高于全省平均水平。全面落实“4321”政银担合作风险分担机制，大力推广“税融通”业务。宣城经济技术开发区7亿元小微企业集合债券、市国投公司24亿元棚户区改造企业债券及28亿元3个专项债券核准发行。强化用地保障，加强闲置低效土地清理处置，力争土地供应率提高10%以上。坚决打赢市区征迁拆违攻坚战，确保完成征地3400亩，征迁2400户、36万平方米。

（二）加快推进工业调整转型升级。制定实施《中国制造2025宣城行动方案》，开展“工业转型升级年”活动。狠抓产业结构调整。加快战略性新兴产业集聚发展，确保产值增长11%左右。大力加强省战略性新兴产业集聚发展基地建设，推进新材料新能源、PCB应用电子、特种装备制造、节能机电等产业基地申报全省第二批基地。加快传统产业提升改造，支持220户重点企业开展技术改造，完成技改投入300亿元。选择60家企业推广应用工业机器人900台，实施10个智能装备或智能车间项目。采取兼并重组、关停并转等方式，积极稳妥化解169户特困企业过剩产能。培育壮大企业实力。实施企业升级工程，力争新增规模企业120户、产值超亿元企业20户、超10亿元企业3户、百亿元企业1户。加快实施企业上市“翱翔计划”，新增上市（挂牌）企业10家以上，后备企业资源库超过100家。提升科技创新能力。加强高新技术企业培育，新认定高新技术企业40家，高新技术产业增加值增长12%以上。建立省级以上创新平台10家以上，规模工业企业研发机构覆盖率超20%。加强产学研合作，对接合作项目200个以上。支持全社会发明创造，新增授权发明专利超过200件。通过天使基金、自主创新专项资金，引导全社会加大研发投入。

引进高层次创新团队3个。深化质量宣城建设，新增中国驰名商标1—2件、省著名商标24件、安徽名牌产品12个以上。突出开发区转型发展。宣城经济技术开发区重点发展新材料新能源、装备制造、汽车及零部件三大产业，加快推进中兴、中集、中鼎、北斗应用等项目建设,力争新开工工业项目22个、竣工投产20个，亿元以上项目实际到位省外资金50亿元、实际利用外资8900万美元。支持广德开发区争创国家级开发区，加快“筹”字号省级开发区“转正”步伐。全力推进大众创业。宣传企业家创业事迹，大力弘扬企业家精神，鼓励企业加快发展。强化创业培训，激发创业创新活力，加快建设创业园区和基地。深化商事制度改革，推进工商注册制度便利化，新登记私营企业3600户、个体工商户1.4万户。

（三）加快中心城市建设。坚持科学规划引领。以新一轮城市总体规划为指导，优化市域产业、人口和基础设施布局。加强城市功能规划，编制完成城市综合交通体系、地下空间暨人防工程综合利用等专项规划。推进市县“多规合一”。强化建筑色彩、景观轴线、节点地标的高水平城市设计，塑造城市特色。加强“一山两湖两江六河”及周边地区规划管控，严格规划刚性约束。全面提升建设水平。深入开展“城市建设提升年”活动，加快中心城区171个项目建设，确保完成投资120亿元、力争150亿元。推进海绵城市、地下综合管廊建设。实施老城区道路畅通工程三年计划和市区公共自行车一期项目。启动敬亭山至梅溪路城市绿轴、宛溪河历史文化景观长廊、体育学校、游泳馆、消防训练基地、警察学校、谢朓历史文化公园、青弋江大道六标等项目建设，加快北门老城区、宛溪河综合整治（二期）及周边棚户区改造、水阳江大道闭合段等项目建设，建成植物园、残疾人康复中心、敬亭圩污水处理厂二期和防震减灾综合业务用房及测震台等项目，图书馆、规划馆、博物馆新馆全面开馆使用。强化城市服务和管理。推动以人为核心的新型城镇化，积极推进农业转移人口市民化，加快城镇常住人口基本公共服务均等化。探索建立棚户区改造和商品房市场联动发展机制，积极推行征迁项目货币化安置，加快房地产去库存。科学布局城市商业网点，新建万达广场等项目，推进中央商务区商圈建设，培育发展特色商业街区，完善社区商业便利消费体系。加快智慧城市建设，规范运行市社会服务管理信息化平台，推进人口、法人、空间地理三大基础数据库建设，扩容政务云计算中心，建成数据交换平台。改进城市管理工作，实施市容管理和执法能力提升工程，健全城市网格管理机制，持续开展“三车”、背街后巷等集中整治。打造综合交通网络。继续开展“交通建设会战年”活动，完成交通投资60亿元。加快宣狸、广宁等高速公路建设进度，完成溧广高速和鸿门互通工程，新增高速公路里程39公里。全力推进芜黄高速宣城段前期工作。推进G318宣城至十字铺等5条一级公路建设。开工建设绩谭旅游快速通道。加快农村公路畅通工程建设。抓好水阳江航道整治和宣州综合码头工程建设。做好商合杭、皖赣铁路宣城至绩溪段扩能改造服务保障，推进杭黄铁路和巷口桥货场建设，完成市客运站和宣城火车站东站房主体工程。加强水利电力等基础设施建设。加快水利建设步伐，争取水阳江中游治理和凤凰山水库跻身国家投资规划，加快推进港口湾水库灌区、牛岭水库等重大项目前期工作，开工建设青弋江综合治理、扬溪源水库和水阳江生态蓄水等工程。深入推进水利工程建后管护体制机制改革，抓好水生态文明城市试点工作。开工建设准东—华东（皖南）±1100千伏特高压直流输电工程，继续加强主电网工程建设。启动4G+网络工程，加强农村光网建设。

（四）提速发展现代服务业。大力发展文化旅游产业。全面实施文化旅游产业“八大工程”，加快市本级“两轴三片区”建设，力争文化产业增加值和旅游收入分别增长10%和18%左右。启动国家级全域旅游示范区创建。大力推进敬亭山旅游度假区5A创建，启动门票收费管理和茶香小镇等项目前期工作，加快中华诗词文化博览园、林相改造、景区基础设施提升等项目建设。支持大桃花潭、太极洞、徽杭古道等景区创建5A，力争新创4A级以上景区2—3家。启动老旧景区提升改造工程，加快乡村旅游基础设施建设。举办“多彩宣城—宛陵湖美丽之夜”全国露营帐篷节。创成中国书法城、中国摄影之乡。加快宣城文教创意产业园建设，推进安徽地产品直销中心建设。促进生活性服务业升级。发挥新消费引领作用，重点发展健康、养老、体育、法律、教育培训等生活性服务业。加快建设以居家为基础、社区为依托、机构为补充的多层次养老服务体系，大力发展中医医疗服务、中医药养生、健康旅游等健康产业，打造“健康宣城、养生福地”。牵头创办（中国）古道联盟，推进徽杭古道、吴越古道、鸦山古道、旌歙古道、水墨汀溪等户外运动基地建设，与黄山等市联合打造国家体育产业基地。积极发展场馆服务、体育培训、体育传媒等新兴产业。完善商贸流通体系，改造提升批发零售、住宿餐饮等传统服务业。大力培育商贸流通企业，力争新增限额以上商贸企业15家以上。加快生产性服务业发展。以产业转型升级需求为导向，重点发展现代金融、现代物流、信息技术服务、电子商务和人力资源服务等。大力发展普惠金融，设立旌德村镇银行。引进金融租赁、融资租赁、消费金融和财务公司等新型金融机构，稳妥发展互联网金融等新兴金融业态。加强金融生态环境建设，打击恶意逃废债行为和非法集资。大力发展农村电子商务。加快宣城现代服务业产业园区建设与发展，确保新开工项目8个、投产5家。

（五）加快开放合作发展。构建区域合作新格局。围绕打造长江经济带重要战略支撑，抓好承接产业转移示范区建设。推进落实与南京、杭州签署的战略合作框架协议，加快合作共建园区建设。深入推进招商合作。完善招商招才引技机制，健全招商联动机制和考核评价体系，建立招商引资政策“一单清”。聚焦主导产业、战略性新兴产业和国内外500强企业，开展精准招商。深化产业研究，加强重大招商项目的谋划和编制，深化与央企、知名民企、台企对接，拓展境外招商，强化市场化招商和孵化器招商，力争亿元以上项目实际到位省外资金增长10%以上、新引进单体投资5亿元以上项目35个左右、新建项目主导产业契合度达50%。大力发展对外贸易。继续实施外贸“双百工程”，引进和培育外贸综合服务企业，着力打造食品、农产品出口基地，大力推进宣城口岸建设，统筹协调推动企业“走出去”，争取安徽皖东南保税物流中心和市区公用型保税仓库获批，鼓励进口先进技术、装备、原材料和消费品，提升进口比重。

（六）发展壮大现代农业。培育新型农业经营主体。全面完成农村土地承包经营权确权登记颁证工作，积极推进全国集体林业综合改革试验示范区建设和供销合作社综合改革，深化农村集体资产产权制度改革，扩大农民土地股份合作社试点，加快推进农村产权交易市场建设。依法推进土地经营权有序流转，加快适度规模经营步伐。培育现代农业产业化联合体80个，其中省级6个以上。促进农民专业合作社、家庭农场规范化发展，新增省级示范家庭农场40个、农民专业合作社示范社7个，培育新型职业农民2500名。推进生态农业建设。积极争创省级现代生态农业示范市、县。开展生态高标准农田建设试点，新增高标准基本农田10万亩。加强农业投入品监管，实施化肥、农药零增长计划。加大规模养殖场的污染治理，提高废弃物综合利用能力。创建省级农产品质量安全县。推进农产品质量追溯体系建设。加快农机化发展，提高农作物耕种收综合机械化水平。推进皖南特色农业气象服务工程建设。调整优化农业结构。稳定粮食种植面积，开展高产创建和专有品牌粮食试点。推进“粮安工程”建设，加快“放心粮油”和“主食厨房”工程进度。继续实施茶产业提升计划，新改建良种茶园1.5万亩、低产茶园2.5万亩以上。支持畜禽业健康发展，推进畜禽良种工程建设和标准化规模养殖，新增省级以上畜禽标准化养殖小区4个以上，创建农业部水产健康养殖示范场2家。推进竹产业、木本油料产业、林下经济等“富民工程”，实现林业总产值460亿元。大力发展农产品加工。加快现代农业园区建设，扶持重点龙头企业发展，延伸产业链，开发优质安全产品，认证农业“三品一标”40个，力争农产品加工产值达735亿元。

（七）进一步营造宜居宜业环境。扎实推进“六城同创”。深入推进全国文明城市创建，组织开展文明创建提升年活动，大力实施七大提升行动，不断提升市民文明素质和城市文明程度，推进志愿服务常态化和制度化建设，申创全国未成年人思想道德建设工作先进城市。开展国家生态市创建攻坚，完成国家环保模范城市技术评估和考核验收。巩固国家园林城市、森林城市创建成果，全面实施绿化提升行动，新增220万、改造提升134万平方米绿化面积；新造林 7.29万亩。深入实施国家生态文明先行示范区建设，积极开展全国智慧城市、海绵城市、卫生城市、历史文化名城等创建工作。加大环境治理力度。实行最严格的水资源管理和环境保护制度。深入实施大气、水、土壤污染防治行动计划，实行重点企业排污许可证制度，健全重点流域跨界环境污染联防联控机制，加强城镇生活饮用水水源保护。推进城乡环境保护一体化，全面构建市县乡村四位一体环境监管体系，继续开展环保“绿剑行动”，严厉查处环境违法行为。推进美丽乡村建设。继续推进中心村建设三年全覆盖工作，优先开展乡镇政府驻地所在行政村中心村建设，同步推进环境整治。完成“三线四边”环境治理任务，推进农村清洁工程市场化改革，全面开展农村生活垃圾分类减量处理，巩固完善农村环境治理常态长效机制，全面改善农村人居环境。

（八）深入推进共享发展。坚决打赢脱贫攻坚战。坚持精准扶贫、精准脱贫，建立健全精准扶贫台账和“单位包村、干部包户”制度，深入实施脱贫攻坚八项行动，确保完成7.35万建档立卡贫困人口在现行标准下全部脱贫的年度攻坚任务。拓展实施民生工程。继续实施33项民生工程，强化建后管养和绩效评估，认真办好市政府民生新实事，确保民生支出比重达80%以上。重点抓好高校毕业生和就业困难群体就业。做好社会保险扩面征缴工作，提高城乡低保水平，达到兜底脱贫标准。加大城镇棚户区和城乡危房改造力度，完成棚户区改造5411套、农村危房改造6500户。完成农配网改造升级投资11.9亿元，解决低电压用户4.3万户。提升发展各项事业。启动实施社会事业发展三年行动计划。全面改善薄弱学校办学条件，加快推进义务教育均衡发展，确保绩溪、旌德、泾县义务教育发展基本均衡县通过国家认定。完成学前教育二期行动计划。积极推进普通高中改革发展。推进职业教育教学改革、校企合作、中高职衔接。编制宣城职教园区规划。支持宣城职业技术学院建设地方技能型高水平大学。继续实施特殊教育提升计划，建成宁国、泾县特教中心。实施乡村教师支持计划，推进教师无校籍管理改革。加快国家教育装备综合改革实验区建设。继续实施基础教育信息化建设工程。举办宛陵湖半程马拉松、全国乒乓球业余联赛等体育比赛，承办2016年世界男排联赛分站赛和全国山地自行车冠军赛。积极开展全民健身活动，试行“医保健身一卡通”和发放特定人群体育消费券工作。推进省级乡镇文化服务中心试点和农民文化乐园建设，启动社区综合文化服务中心建设。开展宣城春晚、三月三诗歌吟唱会、宛陵湖文化艺术节、皖南花鼓戏寻根调研等群众文化活动。推进乡村应急广播网建设。加强对传统村落和古民居的保护。开展“非遗”进学校、进社区、进景区等活动。深化医药卫生体制综合改革，积极探索“3+3”分级诊疗模式。完成宣城中心医院搬迁，新建市妇幼保健院，支持市人民医院创建三甲医院。加快推进社区卫生服务中心规范化建设，加强公共卫生服务和卫生应急体系建设。推进市救助站新站、精神病人福利机构和精神病院建设。依法实施“全面两孩”政策，健全完善人口计生利益导向机制；全面实施“健苗工程”新生儿遗传代谢出生缺陷疾病干预救助项目，保持出生人口性别比平衡。确保马山生态陵园殡仪馆投入使用，完成县乡公益性公墓建设任务。积极做好第三次全国农业普查准备工作。积极创新社会治理。全面推进社会信用体系建设，建成公共信用服务平台。深化社区体制改革，提升社区服务水平，开展农村社区建设试点。启动“七五”普法，全面推进依法治市，继续开展机关集中学法月活动。推进公共法律服务体系建设，提高法律援助保障能力。推广仲裁解决民商事纠纷机制，吸纳社会力量参与社区矫正和安置帮教工作。大力推进“铸安”行动，健全安全生产、反恐防控“党政同责、一岗双责、失职追责”的责任体系，推动企业主体责任落实，有效预防较大及以上事故发生。全力争创食品药品安全城市和食品安全示范县。推进社会服务管理信息化建设，加强基层综治维稳信访工作中心（站）建设。健全社会稳定风险评估机制，深入推进矛盾纠纷排查化解和重点地区整治工作，加强行业性专业性调解组织和社会调解组织建设，健全完善数字化立体化社会治安防控体系。加强突发事件应对处置能力建设。继续落实领导开门接访制度，拓展信访信息系统功能，提升信访事项的及时受理率、按期办结率和群众满意率。积极培育发展社会组织。支持工会、共青团、妇联等人民团体参与社会治理。加强人民防空建设，推进军民融合深度发展，巩固提升全国双拥模范城创建成果。

四、全力推动政府效能实现新提升

全面建成小康社会，开创“十三五”发展新局面，政府必须不断加强自身建设。我们将按照“四个自觉”的要求，高标准践行“三严三实”，进一步加强政府系统党的建设，勇担富民强市的历史使命，朝夕勤勉、永不懈怠，进一步提升政府工作效能，切实增强政府公信力和执行力，努力建设人民满意政府。

大力开展创新型政府建设。强化创新理念，大力崇尚创新，将创新型政府建设作为基本立足点，坚持上下联动、全员参与，坚持问题导向和目标导向相统一，精心设计创新项目，强力实施政府工作创新工程。抓好政策学习和解读，强化经济形势分析研判，密切关注新情况、新变化，改进调查研究，创新工作举措，破解发展难题和突出矛盾。加强政府工作方式与市场接轨，改善政策杠杆引导，推动由扶持企业向扶持产业转变。抓好产业投资基金运行，落实支持项目，加强对主导产业及重点发展产业的定向扶持，进一步增强工作实效。

深入推进政府系统改革。深化政府职能转变和机构改革，推进事业单位分类改革，建立功能明确、治理完善、运行高效、监管有力的管理体制和运行机制。持续推进简政放权，规范完善政府权力清单和责任清单运行机制，严格落实涉企收费清单制度，探索实施负面清单制度，强化事中事后监管。适应供给侧改革要求，推动资源要素优化配置，落实“去产能、去库存、去杠杆、降成本、补短板”五大任务，从源头上改善、提高企业生产效率和产品质量。健全政府职责体系，严格执行“四项制度”，完善绩效管理和考核评价体系，强化担当意识，强化督查督办，坚决查处有令不行、有禁不止和庸政懒政怠政行为，确保政令畅通。

强化服务型政府建设。增强主动服务意识，在解决群众所忧、满足群众所盼上，下更大气力，谋更多福祉。认真落实密切联系群众的各项制度，深入一线、深入基层，察实情、办实事、求实效。切实提高服务质量，建立完善政府分级帮扶、分层联系企业制度，全力帮助企业降低成本，提高服务的针对性和实效性。持续推进政务服务标准化建设，提升服务水平。深入开展百姓问政、百姓热线、政民互动等活动，坚持和完善重要会议邀请“两代表一委员”和市民、网民代表列席制度，切实解决群众反映的突出问题，让群众更好地监督政府。

进一步打造法治政府。把遵守宪法和法律作为政府工作的根本原则，严格按照法定权限和程序行使权力、履行职责。坚持重大事项向市委报告制度，自觉接受人大法律监督、工作监督和政协民主监督以及社会监督。广泛听取民主党派、工商联、无党派人士和人民团体意见。坚持市长领衔办理制度，认真办理人大代表建议和政协委员提案。提高政府立法立规质量，出台政府规章制定程序规定。坚持依法科学民主决策，规范行政决策程序，完善合法性审查和风险评估机制，实行重大决策终身责任追究制度及责任倒查机制。全面推行政府法律顾问制度，充分发挥人民监督员作用。深化政务公开，加强公开载体和平台建设。

加强廉洁政府建设。带头严守政治纪律和政治规矩，严格落实党风廉政建设主体责任。深入贯彻中央八项规定和省、市委“三十条规定”，驰而不息加强作风建设，严肃查处损害群众利益的突出问题。紧盯“四风”问题新形式、新动向，加大明查暗访力度，注重从廉政建设巡查、责任制检查考核、审计监督等工作中发现问题线索，加大问责力度，强化巡查成果运用。加大审计监督力度，实现对公共资金、国有资产、国有资源和领导干部履行经济责任情况审计全覆盖。全力推进预算管理改革，加强政府资金使用与管理，严格控制“三公”经费支出。

各位代表，回顾“十二五”光辉历程，我们满怀豪情；创造“十三五”新的未来，我们信心倍增！雄关漫道真如铁，而今迈步从头越。让我们在市委的坚强领导下，紧紧团结和依靠全市人民，万众一心，扎实工作，顽强拼搏，锐意进取，为提前全面建成小康社会、共同创造“十三五”宣城新的辉煌而努力奋斗！
